# Integrating Artificial Intelligence and Automation Into Living Guideline Development: A Scoping Review of Existing Frameworks, Methods, and Applications

**DOI:** 10.1002/gin2.70079

**Published:** 2026-07-06

**Authors:** Nofisat Ismaila, Syed Arsalan Ahmed Naqvi, Lawrence Mbuagbaw, Jinhui Ma, Lehana Thabane

**Affiliations:** ^1^ Department of Health Research Methods, Evidence, and Impact McMaster University Hamilton Ontario Canada; ^2^ American Society of Clinical Oncology Alexandria Virginia USA; ^3^ Division of Hematology and Oncology Mayo Clinic Phoenix Arizona USA; ^4^ Department of Anesthesia McMaster University Hamilton Ontario Canada; ^5^ Department of Pediatrics McMaster University Hamilton Ontario Canada; ^6^ Biostatistics Unit, Father Sean O'Sullivan Research Centre, St Joseph's Healthcare Hamilton Ontario Canada; ^7^ Centre for Development of Best Practices in Health (CDBPH) Yaoundé Central Hospital Yaoundé Cameroon; ^8^ Division of Epidemiology and Biostatistics, Department of Global Health Stellenbosch University Cape Town South Africa; ^9^ St Joseph's Healthcare‐Hamilton Hamilton Ontario Canada; ^10^ Faculty of Health Sciences University of Johannesburg Johannesburg South Africa

**Keywords:** artificial intelligence, automation, frameworks, living guidelines

## Abstract

**Background:**

Artificial intelligence (AI) and automation offer opportunities to enhance the efficiency, timeliness, and sustainability of living guidelines (LGs). However, how AI and automation have been integrated into existing LG development frameworks remains unclear. This scoping review represents the first step in a broader programme of work aimed at developing a framework which aims to guide responsible and coordinated AI adoption across all phases of LG development.

**Objective:**

To identify existing frameworks, methods, and approaches that integrate AI or automation into any stage of LG development.

**Methods:**

We conducted a structured search of PubMed, Embase, Web of Science, Scopus, Cochrane Database for Systematic Review and Cochrane Central Register of Controlled Trials from inception to March 12, 2026. We included peer‐reviewed articles describing frameworks, models, or methods that applied AI or automation in the development of LGs. We extracted data into a standardised form, capturing study characteristics, AI methods, targeted guideline processes, and key findings and synthesised findings using a thematic narrative approach.

**Results:**

Of 1090 records identified, three studies met the inclusion criteria. The included studies described AI or automation applied to select components of the LG process, to support continuous evidence surveillance, to semi‐automate study screening, and incremental updating of living systematic reviews. None addressed multiple stages of LG development in an integrated manner.

**Conclusions:**

Current evidence demonstrates fragmented and narrowly focused AI or automation applications within LG development processes. Our findings highlight opportunities for future work to develop and evaluate more comprehensive frameworks that span multiple stages of the LG lifecycle.

## Summary

- ***What is already known on the subject***:
  ◦ Living guidelines (LGs) rely on continuous evidence surveillance, rapid synthesis, and iterative updating, creating substantial methodological and operational demands on resources.
  ◦ Artificial intelligence (AI) and automation have shown promise in supporting discrete evidence synthesis tasks such as literature monitoring, study screening, synthesis and automated review updates.
  ◦ No established, comprehensive framework currently exists to guide integration of AI or automation across the full LG development cycle.
- ***What this study adds***:
  ◦ This scoping review identified only three studies that described frameworks or methods integrating AI or automation into any part of the living evidence or LG ecosystem.
  ◦ These studies focused on isolated components within the LG development like continuous evidence surveillance, semi‐automated screening, and incremental living systematic review updating, rather than offering end‐to‐end guidance for LG development.
  ◦ This review reveals substantial gaps in governance, transparency, standards, quality assurance, and real‐world evaluation of AI‐enabled processes within LG development.
- ***Implications for practice, research, and policy***:
  ◦ Guideline developers currently lack structured, comprehensive resources to support safe, transparent, and systematic adoption of AI or automation in LG workflows.
  ◦ The absence of guidance related to governance, oversight, transparency, and quality assurance in included studies highlight important areas for policy makers and methodological organisations to prioritise, particularly as AI‐enabled tools become more widely adopted in LG development.
  ◦ Our findings underscore the need for further development and evaluation of frameworks that link AI and automation applications across the LG development cycle with explicit attention to implementation and methodological rigour.

## 1 Introduction

Living Guidelines (LGs), defined as an optimisation of the guideline development process to allow updating of individual recommendations as soon as or shortly after new relevant evidence becomes available [1], represent a fundamental shift in the typical guideline development process. Despite the potential of LGs, their sustainability and responsiveness are limited by manual processes and lack of integration with digital systems. The increasing complexity and volume of clinical evidence have generated interest in applying artificial intelligence (AI) and automation to key processes in guideline development.

This has led to rapid advances in AI applications in evidence synthesis and guideline development that encompass a range of computational approaches, including machine learning, natural language processing (NLP), and, more recently, large language models (LLMs). While earlier AI‐supported tools focused on classification, pattern recognition, and rule‐based automation (e.g., screening or data extraction), LLMs represent a newer class of generative AI systems capable of synthesising information, drafting structured summaries, supporting recommendation formulation, and facilitating interactive decision support. The rapid evolution and increasing accessibility of LLM‐based systems have intensified interest in their potential role across multiple phases of LG development [2].

Furthermore, automation, which can be rule based (non‐AI) or AI‐powered methods, uses these technologies to execute components of guideline development workflows with varying levels of human involvement (e.g., automated evidence screening, data extraction). Thus, AI and automation are usually applied along a continuum, from rule‐based assistance to more adaptive, data‐driven approaches. Together, these technologies offer opportunities to reduce time, labour and resource demands on guideline developers while supporting more timely and sustainable LG development processes [3, 4].

However, these innovations have largely been applied in isolation. The LG development process typically comprises several phases: protocol development, systematic review or evidence synthesis, guideline content generation, dissemination and monitoring [5]. Therefore, a comprehensive framework for integrating AI and automation into LG development should encompass most, if not all, of these components to ensure efficiency, transparency, and consistency across the LG lifecycle. Embedding AI throughout these phases can enhance evidence surveillance, streamline data synthesis, support dynamic recommendation updates, and facilitate continuous monitoring and dissemination of guidance in near real time.

Given the breadth and complexity of the LG development lifecycle, guidance from related evidence‐based initiatives offers useful context for how AI may be responsibly integrated into such processes. Several initiatives and methodological efforts provide valuable context for responsible and sustainable AI use in evidence‐based processes. These include the *Responsible AI in Evidence SynthEsis (RAISE)* collaboration, led by Cochrane, the Campbell Collaboration, the Joanna Briggs Institute, and the Collaboration for Environmental Evidence [6]; the Guidelines International Network (GIN) McMaster Guideline Development Checklist Extension for Integrating Artificial Intelligence in the Guideline Enterprise (Guidelines‑Artificial Intelligence Extension) [7]; the GIN principles for trustworthy use of AI in guideline development [8] and the International Consensus Guideline for Trustworthy and Deployable Artificial Intelligence in Healthcare (FUTURE‐AI Consortium) [9]. While these initiatives do not specifically focus on LG development, they offer foundational principles related to governance, transparency, and implementation that can inform the development of comprehensive frameworks for integrating AI and automation into the LG processes.

The extent to which these existing guidance have been translated into frameworks or methods specific to LG development remains unclear. As such, this scoping review aims to identify and characterise existing frameworks, models, and methodological approaches that apply AI and/or automation to any phase of LG development, and to synthesise key gaps and areas for future methodological development that can inform the design of more integrated LG frameworks.

## 2 Methods

This scoping review is reported according to the Preferred Reporting Items for Systematic Reviews and Meta‐analysis checklist for scoping reviews (PRISMA ‐ ScR) [10]. The protocol for this review was registered on the Open Science Framework (Registration ID: p8b56).

### 2.1 Literature Search

We searched electronic databases, which include PubMed, Embase, Web of Science, Scopus, Cochrane Database for Systematic Review and Cochrane Central Register of Controlled Trials, using a structured search strategy from each database's inception through March 12, 2026. A medical librarian developed the search strategy with input from the principal investigator using controlled vocabulary with supplemental keywords. We limited results to English language publications and did not restrict the publication year or study design. Full search strategies, including controlled vocabulary and keywords, are provided in Supporting Appendix A.

For the purposes of this review, a framework was defined as a structured model outlining components, processes, or relationships guiding AI or automation use within LG development, while a method refers to a specific procedural or technical approach applying AI or automation to a defined LG development related task.

### 2.2 Eligibility Criteria

#### 2.2.1 Inclusion

- Peer‐reviewed articles published in English.
- Studies whose primary aim was to describe, develop, or evaluate a framework, model, or methodological approach that applies AI and/or automation to one or more phases of LG development.
- Studies that explicitly linked AI or automation methods to LG development workflows or closely related living evidence processes (e.g., living systematic reviews used to inform LGs).

#### 2.2.2 Exclusion

- Studies that did not describe the use of AI or automation in any phase of LG development or closely related living evidence processes.
- Articles focused solely on clinical decision support systems, predictive modelling, or AI applications unrelated to guideline or evidence synthesis workflows.
- Opinion pieces, commentaries, editorials, blog posts, or perspective articles without a described framework, method, or evaluative component.
- Conference abstracts without sufficient methodological detail to assess eligibility.

### 2.3 Study Selection

Study selection was conducted using the Living Interactive Evidence (LIvE) platform [11]. We used an innovative dual‐reviewer approach by combining a human reviewer (N.I.) with an AI‐assisted reviewer (GPT‐4). The GPT‐4 model was supplied with a structured prompt incorporating the predefined inclusion and exclusion criteria, and its screening decisions for each record were retrieved and recorded. The human and AI reviewer completed screening independently. Any discrepancies between their decisions were resolved through adjudication by a third reviewer (S.A.A.N.). This hybrid human–AI screening approach was used to enhance screening efficiency and to explore the potential role of AI in supporting evidence identification processes relevant to LG development.

### 2.4 Data Extraction

Data from included studies were extracted by one reviewer (N.I) into a data extraction table in Microsoft word document and audited by a second reviewer (S.A.A.N.). The following study variables were extracted: last name of first author, year of publication, country, study aim, type of AI model used, guideline process targeted, and summary of key findings. The form was reviewed and refined by the study team to ensure clarity and consistency prior to data extraction.

### 2.5 Data Analysis

We synthesized findings using a descriptive thematic approach. Following data extraction, two reviewers grouped reported AI or automation applications according to the stages of the LG development process they targeted. Through iterative discussion between reviewers, recurring patterns across studies were identified and organised into thematic categories. Given the small number of included studies and the scoping nature of the review, no formal qualitative coding or theory‐driven thematic analysis was conducted.

In addition to predefined extraction variables, we documented recurring omissions in areas relevant to AI and automation integration, which informed the identification of evidence gaps.

## 3 Results

**Figure 1:**
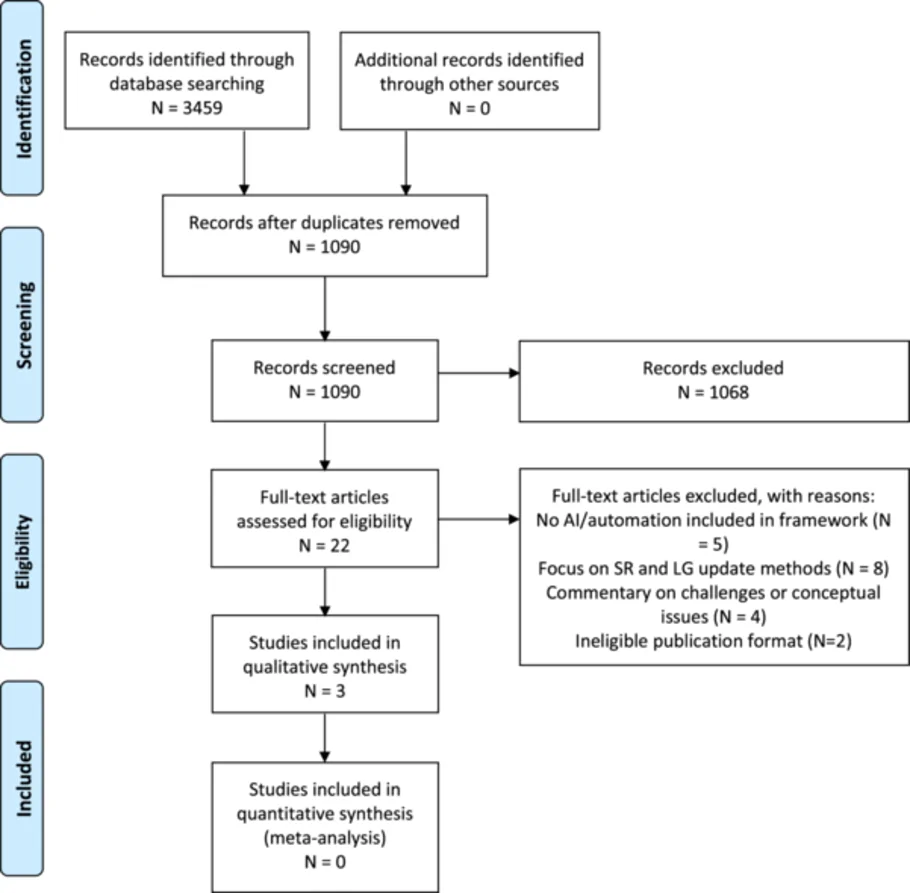
PRISMA flow diagram of literature screening.

The database search yielded a total of 3459 records. After removing duplicates studies (2369), 1090 titles and abstracts were screened. Of these, 22 full‐text articles were assessed for eligibility, and three studies met the inclusion criteria (Figure 1) [12, 13, 14].

The primary reasons for exclusion were: (a) the study did not include AI integration within the framework described; (b) the article focused solely on methods for updating SRs or LGs without presenting an AI‐ or automation‐enabled framework; (c) the paper discussed challenges or conceptual issues in systematic review or LG development without proposing a structured approach or model or (d) study published as an abstract or protocol. The list of excluded studies is provided in the Supplement.

### 3.1 Characteristics of the Included Studies

The three studies that met the eligibility criteria were Kaiser and Miksch [12], Riaz et al [13] and Martins‐Pfeifer et al. [14] These studies discussed frameworks or models of integrating AI and/or automation in the evidence generation, synthesis and maintenance phase of the LG development process.

Kaiser and Miksch presented the LASSIE (modelling treAtment proceSSes using Information Extraction) method for semi‐automatically updating computer‐interpretable clinical practice guidelines which was developed approximately two decades ago (published in 2009). Conducted in Austria, the study aimed to streamline the development of LGs by combining information extraction and document versioning techniques to detect and formalise changes between guideline versions. Using the British Guideline on the Management of Asthma as a case example, the authors demonstrated that their method could automatically inherit unchanged components and identify modified or new sentences requiring re‐modelling, thereby significantly reducing manual workload. While the approach was reported to have successfully improved efficiency and traceability in updating formalised guideline models, it focused primarily on technical modelling rather than the broader LG lifecycle. Nevertheless, this study represents one of the earliest informatics‐based approaches to supporting continuous guideline updating and provides a foundational example of AI‐assisted processes in LG maintenance.

Riaz and colleagues on the other hand propose an AI‐supported, linear, integrated, interactive, and collaborative framework, the living interactive evidence synthesis framework, that enables continuous, real‐time evidence updating as new studies become available, enabling a “living” evidence synthesis [13]. The authors emphasise the interactive interfaces and continuous meta‐analytic recalculations (living meta‐analysis or continuous cumulative meta‐analysis when new evidence becomes available), illustrating how AI can support more responsive and adaptive evidence systems. Although the model was conceived to enhance living systematic review workflows, its components including automated surveillance, semi‐automated screening, structured data synthesis, and interactive updating are directly relevant to the evidence synthesis phase of LG development. A prototype of this framework is being piloted and has been used for several network meta‐analyses in the field of oncology [15, 16].

Martins‐Pfeifer et al. [14] described a structured framework for living guideline development that spans the full guideline lifecycle, including planning, evidence synthesis, recommendation development, dissemination, and implementation. The framework incorporates elements of automation within the evidence synthesis phase, such as AI‐assisted literature monitoring and data extraction, while maintaining human oversight for critical appraisal and decision‐making. Although the specific AI methodologies were not fully detailed, the framework reflects an integrated approach to sustaining continuously updated guidelines and highlights the potential role of automation in supporting key components of living guideline workflows.

All three studies focused on the evidence surveillance and evidence synthesis components of the LG process, with none addressing downstream phases such as recommendation generation, manuscript drafting, or dissemination. Further details of the characteristics of each study are included in Table 1.

**Table 1:** Characteristics of included studies.

Author year country	Aim	AI/Automation component	Type of AI approach	Guideline process targeted	Key findings
Kaiser & Miksch 2009 Austria	To develop and test a semi‐automatic approach (LASSIE) for updating computer‐interpretable clinical guidelines, enabling the creation of “living” guidelines.	Information extraction and text comparison methods to identify and formalise new or changed content in guideline versions.	Knowledge‐based system/rule‐based logic	Updating and maintenance of computer‐interpretable guideline models.	The LASSIE method semi‐automatically detected modified text, inherited unchanged components, and generated structured models, reducing manual workload while maintaining traceability and version control. Demonstrated efficiency and feasibility of automating parts of the guideline updating process
Riaz et al. (2024) USA	To describe the current landscape and propose an AI‐empowered integrated framework for living interactive SR MAs for efficient and scalable evidence synthesis.	Automated evidence surveillance using scheduled database queries (potentially via APIs) combined with machine‐learning–assisted prioritisation of newly retrieved records to support continuous updating of living evidence syntheses	Supervised machine learning classifier (NLP‐based)	Living evidence synthesis & interactive updating	Proposed an AI‐supported, linear, integrated, interactive, and collaborative framework that enables continuous, real‐time evidence updating as new studies become available, enabling a living synthesis
Martins‐Pfeifer et al. (2026) USA	To present the methodology for developing clinical or public health evidence‐informed guidelines, with a specific focus on the living framework for ongoing evidence synthesis and maintaining up‐to‐date recommendations.	AI‐assisted evidence monitoring and data extraction	Supervised NLP/ML‐evidence monitoring	Evidence surveillance and updating	Developed a framework for living guideline development including integration of automation in evidence surveillance and synthesis

Abbreviations: AI, artificial intelligence; ML, machine learning; NLP, natural language processing.

### 3.2 Descriptive Thematic Findings

Based on the three studies identified, the following thematic categories emerged regarding the use of AI or automation in components of the LG development process.

#### 3.2.1 Automation of Evidence Surveillance for Living SRs

Two of the studies highlighted the role of automation in continuous literature surveillance, enabling more timely updating of SR. Martins‐Pfeifer et al. [14] described the integration of automated AI‐assisted literature monitoring and data extraction within a living evidence and guideline's framework, while Riaz et al. [13] described AI‐enabled continuous search optimization that updates the evidence database as new studies become available. This approach combines automated periodic database queries potentially implemented through application programming interfaces (APIs) or scheduled search pipelines with machine learning–assisted prioritisation of newly retrieved records to identify studies most likely to meet inclusion criteria.

#### 3.2.2 Semi‐Automated Screening and Study Selection

Martins‐Pfeifer and Riaz et al. both described approaches in which AI or machine‐assisted algorithms are used to support the screening and study selection stages of evidence synthesis. In these models, AI tools assist by prioritising, classifying, or filtering citations, while human reviewers retain responsibility for verifying inclusion decisions. This hybrid approach, often referred to as “human‐in‐the‐loop” screening, has the potential to substantially reduce the manual workload traditionally required during title and abstract review, particularly in the context of continuous evidence surveillance for living SR. Although neither study provided extensive empirical evaluation, their descriptions illustrate how semi‐automated screening could improve both efficiency and timeliness in the evidence‐processing components of LG development. These examples also highlight the importance of maintaining human oversight to ensure accuracy and mitigate risks associated with automated misclassification.

#### 3.2.3 Machine‐Assisted Data Extraction and Structuring

Kaiser & Miksch proposed the LASSIE model in 2009 which is one of the earliest studies to look into this concept. Developed nearly two decades ago, the model emphasised semi‐automated detection of modified text in guideline manuscripts, tracks unchanged components, supporting dynamic and partially automated guideline development processes. While LASSIE represents an early and conceptually important informatics‐based effort to support dynamic guideline processes, its technical architecture predates the emergence of contemporary machine learning systems and LLMs. As such, its current practical applicability may be limited; however, its conceptual emphasis on structured, computable, and modular guideline representation remains relevant to ongoing efforts in AI‐enabled LG ecosystems. Riaz et al. similarly envisioned the use of novel variant language and LLMs with human oversight supported data extraction in SRs for real time updating.

#### 3.2.4 Real‐Time or Incremental Updating of Evidence Outputs

A shared focus across studies was the concept of incremental updating, whether for literature, data extraction, or meta‐analytic results. Riaz et al. proposed real‐time recalculations, while Martins‐Pfeifer et al., proposes an AI‐assisted living evidence review within a living guideline ecosystem.

#### 3.2.5 Conceptualisation of AI‐Enhanced Evidence Ecosystems

Riaz et al. offered a broader conceptual vision for an integrated, automated evidence ecosystem suggesting how AI could contribute across searching, screening, data extraction, and synthesis. This theme reflects growing recognition of the need for coordinated technology‐supported workflows, even though operationalization remains limited.

### 3.3 Evidence Gaps Identified

**Figure 2:**
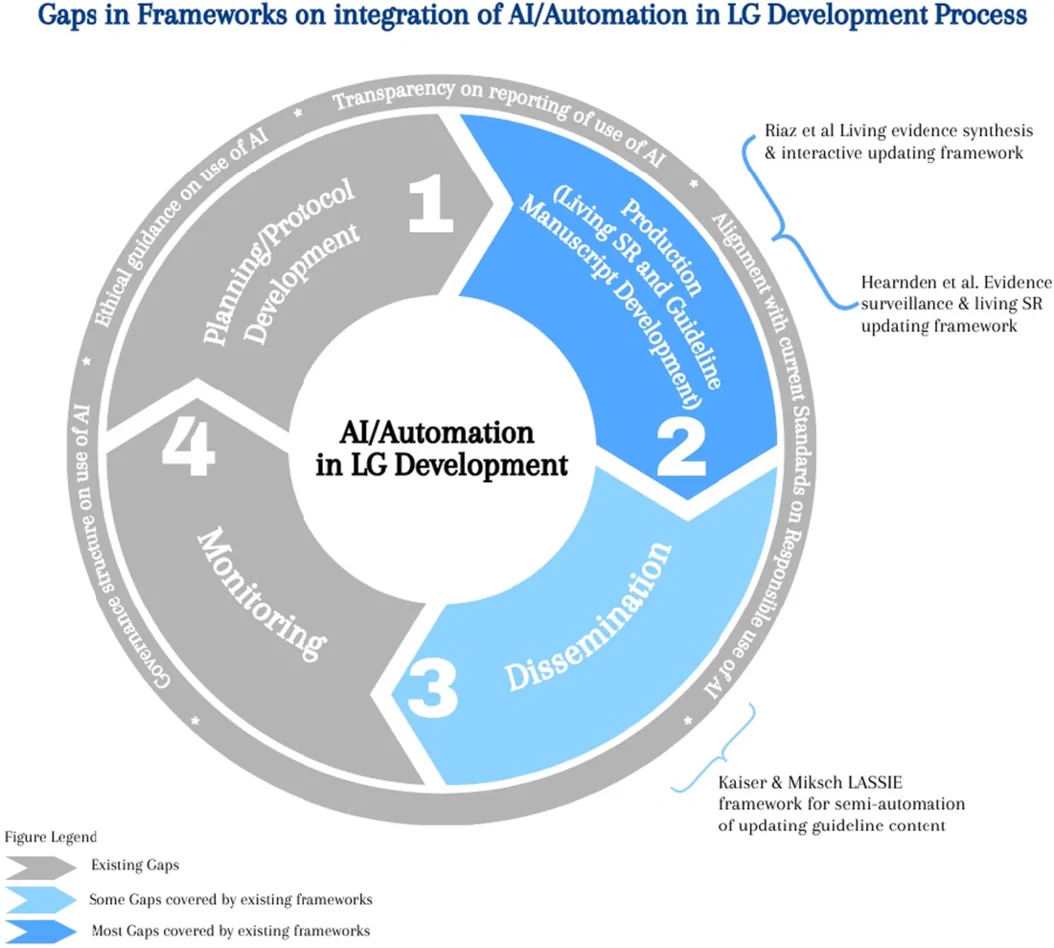
Identified gaps.

The basic framework for developing LGs without AI or automation integration as described by El Mikati and colleagues, describes four major phases including planning, production, reporting, and dissemination [5]. Despite the contributions of the identified studies in this review, they were mostly focused on one of these phases which is the production phase that includes the evidence synthesis stage. Thus, multiple gaps were identified from the result of this review which are critical for a comprehensive framework on integrating AI and/or automation into LG development (Figure 2).

#### 3.3.1 Lack of End‐to‐End Frameworks Covering the Living Guideline Lifecycle

As noted above, none of the included studies presented a framework that integrates AI or automation across the entire LG development cycle. Several critical phases remain unaddressed, such as the planning stage (where AI could support protocol development), the production phase (including recommendation drafting), as well as reporting, dissemination, and monitoring. Each of these stages presents opportunities for meaningful automation or AI‐enabled decision support, yet current efforts remain siloed. This suggests opportunities for future work to explore how these approaches might be coordinated across multiple phases, rather than implemented in an isolated manner.

#### 3.3.2 Lack of Governance, Transparency, and Alignment With Responsible AI Standards

None of the included studies described a governance structure or guidance approach for integrating AI or automation into their processes, nor did they outline how quality control, validation, or bias mitigation were ensured. While Riaz et al. and Martins‐Pfeifer et al. mentioned some level of human oversight, they provided little detail regarding the extent of this oversight, or the roles and qualifications of the individuals involved. Moreover, none of the studies explicitly reported alignment of their methods with established responsible AI use principles, such as the Organisation for Economic Co‐operation and Development (OECD) AI Principles [17], European Union Ethics Guidelines for Trustworthy AI [18], or FUTURE‐AI guidelines [9], which emphasise accountability, explainability, human agency, and risk management. This lack of attention to governance and responsible AI standards represents a critical gap, as these principles are essential to ensuring that AI‐enabled processes in LG development are trustworthy, transparent, and ethically implemented.

#### 3.3.3 Limited Empirical Evaluation of AI Tools in Real‐World Living Guideline Processes

Across the included studies, applications of AI and automation were described largely at a conceptual or prototype level, with no empirical evaluations conducted within real‐world LG development environments. Instead, most evaluations were performed in the context of living SRs. Riaz et al. reported limited pilot testing of their proposed framework on selected living SRs, and Martins‐Pfeifer et al. proposed living guideline framework is yet to be evaluated. Kaiser and Miksch (2009) was the only study to pilot their approach within an LG context, although this was restricted to a single component of the guideline updating process and it's technical architecture might be outdated. As a result, evidence on the practical feasibility, performance, and acceptability of AI‐enabled processes within LG‐producing organisations remains minimal. This lack of real‐world evaluation represents a significant barrier to understanding how these frameworks could be safely, effectively, and sustainably incorporated into LG workflows.

## 4 Discussion

This scoping review systematically mapped the current literature on the integration of AI and automation in LG development processes. Despite growing interest in AI‐enabled approaches within evidence synthesis and guideline development, our review identified only a small number of studies describing structured methods or frameworks relevant to LG workflows. Importantly, the approaches described in these studies were concentrated primarily within the evidence synthesis phase of the LG lifecycle, particularly in areas such as evidence surveillance, citation screening, and incremental updating of systematic reviews. This pattern reflects the broader trajectory of methodological innovation in systematic review automation, where AI tools have historically been developed and evaluated to address labour‐intensive tasks in evidence identification and synthesis [19, 20]. The limited number and narrow scope of identified framework‐oriented studies highlight that, while AI tools are advancing rapidly, the systematic integration of AI and automation across the broader LG development ecosystem, including guideline protocol development, recommendation formulation, dissemination, and implementation monitoring, remains largely underexplored.

### 4.1 Principal Findings

This scoping review identified three studies that explicitly integrated AI or automation into some parts of LG development process. These studies demonstrate the potential for LLM based automation in evidence surveillance, suggesting that AI can enhance the timeliness and efficiency of updates. However, the absence of studies addressing broader, structured integration confirms that this field remains conceptually fragmented and methodologically underdeveloped.

### 4.2 Positioning Within Existing Guidance

As noted in the introduction, several initiatives provide foundational guidance on responsible AI use in evidence‐based processes. Here, we consider how these efforts relate to and help contextualise the findings of this review. While our review identified only three studies directly integrated AI or automation into any part of the LG development cycle, broader initiatives offer important contextual guidance for the use of AI in evidence‐based practice. The RAISE recommendations, for example emphasise that AI tools should complement, rather than replace, human judgment, that developers and users share responsibility for transparency, validation, and governance; and that AI tool deployment should align with established methodological standards. Although RAISE does not specifically address LG development, its principles related to governance, transparency, and accountability offers an essential ethical and procedural foundation for future frameworks [6].

Similarly, the GIN McMaster Guideline Development Checklist Extension for Integrating AI in the Guideline Enterprise (Guidelines‑Artificial Intelligence Extension) represents an effort to extend the widely used GIN–McMaster Guideline Development Checklist (GDC) specifically for integrating AI into the guideline development [7]. This initiative aims to support guideline teams in pre‐planning and transparent reporting of AI use across guideline processes. Although the extension is still under development, its focus on systematic AI integration aligns closely with the objectives of the framework proposed in this work and reflects growing recognition in the guideline community of the need for structured standards when embedding AI.

In addition, there have been publications, consensus documents on principles for trustworthy use of AI in guideline development. The GIN proposes eight broad principles for guideline‐developers using AI (transparency, pre‐planning, additionality, credibility, ethics, accountability, compliance, evaluation) in the guideline enterprise [8]. The International Consensus Guideline for Trustworthy and Deployable Artificial Intelligence in Healthcare (FUTURE‐AI Consortium) defined six guiding principles for trustworthy medical AI (fairness, universality, traceability, usability, robustness, explainability) and 28‐30 best practices across the AI tool lifecycle [9]. The OECD‐AI Guidelines for Development of Trustworthy AI tool, while not specific to healthcare, provides general frameworks for AI governance that includes cross‐cutting elements (fairness, transparency, accountability) relevant to any AI‐integration framework [17].

While all these guidance documents and tools do not focus specifically on LGs, integrating these responsible and trustworthy use principles into LG processes will be critical for ensuring trust, reproducibility, and sustainability in AI and automation enabled LG evidence ecosystems.

### 4.3 Implications for Framework Development

An important consideration is whether LGs are methodologically distinct enough to warrant tailored AI and automation integration frameworks, or whether existing AI‐enabled approaches for systematic reviews or conventional guideline development are broadly applicable. Many of the AI‐supported frameworks identified in related domains, such as automated screening, data extraction, synthesis and risk‐of‐bias assessment, are indeed transferable to LG processes. In this sense, LGs do not require an entirely separate conceptual paradigm for AI and automation integration. However, LGs are distinguished by their continuous evidence surveillance, iterative updating cycles, dynamic version control, and sustained dissemination infrastructure. These longitudinal and cyclical characteristics introduce additional integration points and governance demands that are less prominent in static guideline models. As such, while existing AI and automation integration frameworks for systematic reviews provide an important methodological foundation, they may require adaptation or extension to fully address the operational, technical, and oversight complexities inherent in the LG ecosystems. This distinction is analogous to how reporting standards such as PRISMA serve as a core framework that can be extended to accommodate specific synthesis models.

The limited number of eligible studies identified in this review reflects the early developmental stage of research on AI integration within the LG ecosystems. The gaps identified underscores the need for a comprehensive, methodologically grounded framework to systematically integrate AI and automation tools across all phases of the LG lifecycle, ensuring a coordinated, transparent, ethical and sustainable approach to LG development and updating.

### 4.4 Strengths and Limitations

A key strength of our review lies in its comprehensive search strategy, which spanned both biomedical and technical databases to capture cross‐disciplinary evidence. This approach was essential given the interdisciplinary nature of AI applications in LG development and helped ensure broad coverage of potentially relevant conceptual and methodological work. Another strength was the use of a transparent, protocol‐informed screening and data‐charting process, consistent with PRISMA‐ScR recommendations, which supports reproducibility and methodological rigour. However, several limitations should be noted. The restriction to English language publications may have excluded emerging work in non‐English literature, particularly given the rapid global development of AI‐enabled tools and platforms. Additionally, the emerging nature of the topic likely contributed to the limited yield. Many innovations in AI for evidence synthesis are still in prototype stages, described in grey literature, or not yet formally published. As with many scoping reviews, no formal quality appraisal was conducted, consistent with PRISMA‐ScR guidance. This limits the ability to comment on the methodological robustness or comparative performance of the tools described. Also, given the small number of included studies, thematic findings should be interpreted as descriptive patterns rather than exhaustive categories. Finally, because automation and AI innovations evolve rapidly, it is possible that relevant studies were published after the search was completed, underscoring the need for periodic updating of this review.

### 4.5 Future Directions

In emerging methodological areas, scoping reviews play an important role in systematically mapping the extent of existing work and identifying critical gaps that can guide future research and framework development. While the identified studies focused on discrete steps such as evidence surveillance and screening, it is important to recognise that these frameworks demonstrate transferable characteristics and are likely applicable to LG processes without requiring re‐validation. The question, therefore, is less about whether individual AI frameworks perform adequately within LGs, and more about how these frameworks are embedded within the broader, iterative ecosystem of recommendation development, governance, and dissemination. Recognising these synergies and distinctions helps situate LG‐specific framework development as an issue of coordinated integration rather than isolated framework validation.

The findings from our scoping review provide a clear foundation for such subsequent methodological work. The identified evidence gap will be used to inform the next steps in our project which is to develop a framework that integrates AI and automation in the LG development ecosystem through expert consultation, including a Delphi process, to establish consensus on core components and scope. Future research should also focus on piloting and evaluating the framework in real‐world LG settings, with attention to usability, ethical considerations, governance, and implementation feasibility across different organisational contexts.

## 5 Conclusion

Despite extensive searching, only three eligible studies were identified, confirming that research on integrating AI and automation into LG development is in its infancy. The limited evidence base validates the need for developing structured, transparent, and expert‐informed frameworks to guide future innovation in this area.

## Author Contributions

**Nofisat Ismaila:** conceptualisation, data curation, formal analysis, investigation, methodology, project administration, resources, software, visualisation, writing – original draft, writing – review and editing. **Syed Arsalan Ahmed Naqvi:** conceptualisation, data curation, project administration, resources, software, writing – review and editing. **Lawrence Mbuagbaw:** supervision validation, writing – review and editing. **Jinhui Ma:** supervision validation, writing – review and editing. **Lehana Thabane:** supervision validation, writing – review and editing.

## Funding

The authors have nothing to report.

## Ethics Statement

The authors have nothing to report.

## Conflicts of Interest

The authors declare no conflicts of interest.

## Supporting information

Supporting File 1

Supporting File 2

## Data Availability

The data that supports the findings of this study are available in the supporting material of this article. The data supporting the findings of this study are available from the corresponding author upon request.
