# Supplementary material for: Integrating Artificial Intelligence and Automation Into Living Guideline Development: A Scoping Review of Existing Frameworks, Methods, and Applications
Source: Clin Public Health Guidel. 2026 Jul 6;3(3):e70079. doi: 10.1002/gin2.70079 (PMC13563327; doi:10.1002/gin2.70079)
Supplement: Supplementary file 1 [file GIN2-3-e70079-s001.docx]

**Integrating Artificial Intelligence into Living Guideline Development: A Scoping Review of Existing Frameworks, Methods, and Applications - Supplement**

**APPENDIX A**

**Search strategy**

**PubMed**

Search date: 2.3.25

Number of hits: 666

Search terms: (("living guideline*") OR ("living evidence synthes*s")) OR ("living systematic review*") Filters: English

**Embase <1974 to 2025 January 31>**

Search date: 2.3.25

Number of hits: 745

Search terms:

1. living guideline*.mp.
2. living evidence synthes*s.mp.
3. living systematic
4. 1 or 2 or 3
5. limit 4 to english language review*.mp.

**Web of Science**

Search date: 2.3.25

Number of hits: 621

Search terms: "living guideline*" (All Fields) OR "living evidence synthes*s" (All Fields) OR "living systematic review*" (All Fields) Languages: English

**Scopus**

Search date: 2.3.25

Number of hits: 660

Search terms: (TITLE-ABS-KEY ( "living guideline*" ) ) OR ( TITLE-ABS-KEY ( "living evidence synthes*s" ) ) OR ( TITLE-ABS-KEY ( "living systematic review*" ) ) AND ( LIMIT-TO ( LANGUAGE , "English" ) )

**EBM Reviews - Cochrane Central Register of Controlled Trials <December 2024> EBM Reviews - Cochrane Database of Systematic Reviews <2005 to January 29, 2025**

Search date: 2.3.25

Number of hits: 97

Search terms:

1. living systematic review*.mp. [mp=ti, ot, ab, fx, sh, hw, kw, tx, ct]
2. living evidence synthes*s.mp. [mp=ti, ot, ab, fx, sh, hw, kw, tx, ct]
3. living guideline*.mp. [mp=ti, ot, ab, fx, sh, hw, kw, tx, ct]
4. 1 or 2 or 3

**Author Supplied (2)**

| **DATABASE** | **RESULTS** | **DUPLICATES** | **REMAINING** |
| --- | --- | --- | --- |
| PubMed | 666 | 26 | 640 |
| Embase | 745 | 565 | 180 |
| Web of Science | 621 | 568 | 53 |
| Scopus | 660 | 618 | 42 |
| Cochrane | 97 | 37 | 60 |
| Author Supplied | 2 | 0 | 2 |
| **TOTAL** | 2791 | 1814 | 977 |

**APPENDIX B: List of excluded studies**

**Commentary on challenges or conceptual issues**

1. Australian National COVID-19 Clinical Evidence Taskforce**.** Australian National Clinical Evidence Taskforce COVID-19 drug treatment guidelines: challenges of producing a living guideline. *Med J Aust.* 2023;218(2):85–89. doi:10.5694/mja2.52044
2. König J, Brendel J, Bender A, et al. Digital sovereignty with living guidelines for responsible use of generative artificial intelligence. *Informatik Spektrum.* 2024;47:1–10. doi:10.18420/inf2024_52
3. American Society of Echocardiography. The Living Guidelines Initiative: transforming ASE’s guideline processes. *J Am Soc Echocardiogr.* 2022;35(10):1043–1048. doi:10.1016/j.echo.2022.07.002
4. Akl EA, Meerpohl JJ, Elliott J, et al**.** Methods for living guidelines: early guidance based on practical experience. Paper 5: decisions on methods for evidence synthesis and recommendation development for living guidelines. *J Clin Epidemiol.* 2023;156:24–33. doi:10.1016/j.jclinepi.2022.12.022

**Focus on systematic review or guideline updating methods without proposing an automation-enabled framework**

5. Page MJ, McKenzie JE, Bossuyt PM, et al. Extension of the PRISMA 2020 statement for living systematic reviews (PRISMA-LSR): checklist and explanation. *BMJ.* 2024;384:e079183. doi:10.1136/bmj-2024-079183
6. Marshall IJ, Noel-Storr A, Kuiper J, Thomas J, Wallace BC. Novel tools and methods for designing and wrangling multifunctional, machine-readable evidence synthesis databases. *Syst Rev.* 2021;10(1):155. doi:10.1186/s13750-021-00219-x
7. Page MJ, Moher D, McKenzie JE. Tailored PRISMA 2020 flow diagrams for living systematic reviews: a methodological survey and a proposal. *F1000Research.* 2022;11:517. doi:10.12688/f1000research.51723.3
8. Hillier S, Grimmer K, Merlin T, et al. Towards a new model for producing evidence-based guidelines: a qualitative study of current approaches and opportunities for innovation among Australian guideline developers. *F1000Research.* 2019;8:19661. doi:10.12688/f1000research.19661.1
9. Kaiser M, Miksch S. Use of computer-assisted methods to realize the concept of a living systematic review via an online platform. *Value Health.* 2019;22(Suppl 3):S118.
10. Amezcua-Prieto C, Fernández-Luna JM, Huete-Guadix JF, Bueno-Cavanillas A, Khan KS.
    Artificial intelligence and automation of systematic reviews in women’s health. *Curr Opin Obstet Gynecol.* 2020;32(5):335–341. doi:10.1097/GCO.0000000000000643

**Studies excluded due to absence of AI or automation integration**

11. Kunz R, Schünemann HJ, et al. A framework for the development of living practice guidelines in health care. *Ann Intern Med.* 2022;175(8):1154–1160. doi:10.7326/M22-0514
12. Riaz IB, Naqvi SAA, Hasan B, et al. Framework for a living systematic review and meta-analysis for the surgical treatment of bladder cancer: introducing EVIglance to urology. *Ann Surg Open.* 2022;3(1):e146. doi:10.1097/SP9.0000000000000008
13. Lerner I, Pardo-Hernandez H, Alonso-Coello P, et al. How to update a living systematic review and keep it alive during a pandemic: a practical guide. *Syst Rev.* 2023;12(1):44. doi:10.1186/s13643-023-02325-y
14. Wilson MG, Lavis JN, Gauvin FP, et al. A living critical interpretive synthesis to yield a framework on the production and dissemination of living evidence syntheses for decision-making. *Implement Sci.* 2024;19(1):23. doi:10.1186/s13012-024-01396-2
15. Khan AR, Badar F, Khan S, et al. Updated living systematic review and meta-analysis of controlled trials of mesenchymal stromal cells to treat COVID-19: a framework for accelerated synthesis of trial evidence for rapid approval (FASTER Approval). *Stem Cells Transl Med.* 2022;11(12):1345–1358. doi:10.1093/stcltm/szac038
