# Supplementary material for: Integrating Artificial Intelligence and Automation Into Living Guideline Development: A Scoping Review of Existing Frameworks, Methods, and Applications
Source: Clin Public Health Guidel. 2026 Jul 6;3(3):e70079. doi: 10.1002/gin2.70079 (PMC13563327; doi:10.1002/gin2.70079)
Supplement: Supplementary file 2 [file GIN2-3-e70079-s002.docx]

**Integrating Artificial Intelligence into Living Guideline Development: A Scoping Review of Existing Frameworks, Methods, and Applications - Supplement**

**APPENDIX A**

**Search strategy**

**PubMed**

Search date: 3.12.26

Number of hits: 829

Search terms: (("living guideline*") OR ("living evidence synthes*s")) OR ("living systematic review*") Filters: English

**Embase <1974 to 2025 March 12>**

Search date: 3.12.26

Number of hits: 932

Search terms:

1. living guideline*.mp.
2. living evidence synthes*s.mp.
3. living systematic
4. 1 or 2 or 3
5. limit 4 to english language review*.mp.

**Web of Science**

Search date: 3.12.26

Number of hits: 769

Search terms: "living guideline*" (All Fields) OR "living evidence synthes*s" (All Fields) OR "living systematic review*" (All Fields) Languages: English

**Scopus**

Search date: 3.12.26

Number of hits: 816

Search terms: (TITLE-ABS-KEY ( "living guideline*" ) ) OR ( TITLE-ABS-KEY ( "living evidence synthes*s" ) ) OR ( TITLE-ABS-KEY ( "living systematic review*" ) ) AND ( LIMIT-TO ( LANGUAGE , "English" ) )

**EBM Reviews - Cochrane Central Register of Controlled Trials <December 2024> EBM Reviews - Cochrane Database of Systematic Reviews <2005 to March 12, 2026**

Search date: 3.12.26

Number of hits: 111

Search terms:

1. living systematic review*.mp. [mp=ti, ot, ab, fx, sh, hw, kw, tx, ct]
2. living evidence synthes*s.mp. [mp=ti, ot, ab, fx, sh, hw, kw, tx, ct]
3. living guideline*.mp. [mp=ti, ot, ab, fx, sh, hw, kw, tx, ct]
4. 1 or 2 or 3

**Author Supplied (2)**

| **DATABASE** | **RESULTS** | **DUPLICATES** | **REMAINING** |
| --- | --- | --- | --- |
| PubMed | 829 | 98 | 731 |
| Embase | 932 | 745 | 187 |
| Web of Science | 769 | 708 | 61 |
| Scopus | 816 | 769 | 47 |
| Cochrane | 111 | 49 | 62 |
| Author Supplied | 2 | 0 | 2 |
| **TOTAL** | 3459 | 2369 | 1090 |

**APPENDIX B: List of excluded studies**

**Commentary on challenges or conceptual issues**

1. Australian National COVID-19 Clinical Evidence Taskforce**.** Australian National Clinical Evidence Taskforce COVID-19 drug treatment guidelines: challenges of producing a living guideline. *Med J Aust.* 2023;218(2):85–89. doi:10.5694/mja2.52044
2. König J, Brendel J, Bender A, et al. Digital sovereignty with living guidelines for responsible use of generative artificial intelligence. *Informatik Spektrum.* 2024;47:1–10. doi:10.18420/inf2024_52
3. American Society of Echocardiography. The Living Guidelines Initiative: transforming ASE’s guideline processes. *J Am Soc Echocardiogr.* 2022;35(10):1043–1048. doi:10.1016/j.echo.2022.07.002
4. Akl EA, Meerpohl JJ, Elliott J, et al**.** Methods for living guidelines: early guidance based on practical experience. Paper 5: decisions on methods for evidence synthesis and recommendation development for living guidelines. *J Clin Epidemiol.* 2023;156:24–33. doi:10.1016/j.jclinepi.2022.12.022

**Focus on systematic review or guideline updating methods without proposing an automation-enabled framework**

5. Page MJ, McKenzie JE, Bossuyt PM, et al. Extension of the PRISMA 2020 statement for living systematic reviews (PRISMA-LSR): checklist and explanation. *BMJ.* 2024;384:e079183. doi:10.1136/bmj-2024-079183
6. Marshall IJ, Noel-Storr A, Kuiper J, Thomas J, Wallace BC. Novel tools and methods for designing and wrangling multifunctional, machine-readable evidence synthesis databases. *Syst Rev.* 2021;10(1):155. doi:10.1186/s13750-021-00219-x
7. Page MJ, Moher D, McKenzie JE. Tailored PRISMA 2020 flow diagrams for living systematic reviews: a methodological survey and a proposal. *F1000Research.* 2022;11:517. doi:10.12688/f1000research.51723.3
8. Hillier S, Grimmer K, Merlin T, et al. Towards a new model for producing evidence-based guidelines: a qualitative study of current approaches and opportunities for innovation among Australian guideline developers. *F1000Research.* 2019;8:19661. doi:10.12688/f1000research.19661.1
9. Kaiser M, Miksch S. Use of computer-assisted methods to realize the concept of a living systematic review via an online platform. *Value Health.* 2019;22(Suppl 3):S118.
10. Amezcua-Prieto C, Fernández-Luna JM, Huete-Guadix JF, Bueno-Cavanillas A, Khan KS.
    Artificial intelligence and automation of systematic reviews in women’s health. *Curr Opin Obstet Gynecol.* 2020;32(5):335–341. doi:10.1097/GCO.0000000000000643
11. Kozakiewicz T, Wong R, Ghouze Z . Living evidence and gap maps: a scoping review of automation and living mode parameters reported in 44 digital interfaces and their documentation. Journal of Clinical Epidemiology, 2026; 194
12. Song X, Lian Z, Wang R, Li R, Yang Z, Luo X, Feng L, Ma Z, Pu Z, Wang Q, Ge L, Li C, Chen Y, Yang K, Lavis J. The Phases of Living Evidence Synthesis Using AI: Living Evidence Synthesis (Version 1) J Med Internet Res 2026;28:e76130. DOI: 10.2196/76130

**Studies excluded due to absence of AI or automation integration**

13. Kunz R, Schünemann HJ, et al. A framework for the development of living practice guidelines in health care. *Ann Intern Med.* 2022;175(8):1154–1160. doi:10.7326/M22-0514
14. Riaz IB, Naqvi SAA, Hasan B, et al. Framework for a living systematic review and meta-analysis for the surgical treatment of bladder cancer: introducing EVIglance to urology. *Ann Surg Open.* 2022;3(1):e146. doi:10.1097/SP9.0000000000000008
15. Lerner I, Pardo-Hernandez H, Alonso-Coello P, et al. How to update a living systematic review and keep it alive during a pandemic: a practical guide. *Syst Rev.* 2023;12(1):44. doi:10.1186/s13643-023-02325-y
16. Wilson MG, Lavis JN, Gauvin FP, et al. A living critical interpretive synthesis to yield a framework on the production and dissemination of living evidence syntheses for decision-making. *Implement Sci.* 2024;19(1):23. doi:10.1186/s13012-024-01396-2
17. Khan AR, Badar F, Khan S, et al. Updated living systematic review and meta-analysis of controlled trials of mesenchymal stromal cells to treat COVID-19: a framework for accelerated synthesis of trial evidence for rapid approval (FASTER Approval). *Stem Cells Transl Med.* 2022;11(12):1345–1358. doi:10.1093/stcltm/szac038

**Studies excluded due to study design**

18. Hearnden J, Dudoit K, Kim E, et al: PMU118 USE OF COMPUTER-ASSISTED METHODS TO REALIZE THE CONCEPT OF A LIVING SYSTEMATIC REVIEW VIA AN ONLINE PLATFORM. Value in Health 22:S729, 2019
19. Wieland, Victoria L.S.a; Uysal, Daniel MDa; Probst, Pascal MD, MSc, MBAg; Grilli, Maurizio MLISb; Haney, Caelán M. MDd; Sidoti Abate, Marie A.a; Egen, Luisa MDa; Neuberger, Manuel MDa; Cacciamani, Giovanni E. MD, MSce,f; Kriegmair, Maximilian C. MDc; Michel, Maurice S. MDa; Kowalewski, Karl-Friedrich MD, MSca. Framework for a living systematic review and meta-analysis for the surgical treatment of bladder cancer: introducing EVIglance to urology. International Journal of Surgery Protocols 27(2):p 97-103, October 2023. | DOI: 10.1097/SP9.0000000000000008
